# Non-prompt surgery for patients with acute type A aortic dissection without pre-operative shock and malperfusion

**DOI:** 10.3389/fcvm.2022.988179

**Published:** 2022-12-05

**Authors:** Shye-Jao Wu, Ya-Fen Fan, Yu-Chu Tsai, Shen Sun, Chen-Yen Chien, Jiun-Yi Li

**Affiliations:** ^1^Division of Cardiovascular Surgery, Departments of Surgery, MacKay Memorial Hospital, Taipei, Taiwan; ^2^MacKay Medical College, New Taipei, Taiwan

**Keywords:** aneurysm, dissecting, blood vessel prosthesis implantation, stroke, aorta

## Abstract

**Background:**

Acute type A aortic dissection (ATAAD) requires urgent surgical treatment. However, during daily practice, there were some patients with ATAAD sought for medical attention several days after symptoms occurred and some other patients hesitated to receive aortic surgery after the diagnosis of ATAAD was made. This study aims to investigate the surgical outcomes of non-prompt aortic surgery (delayed diagnosis caused by the patient or delayed surgery despite immediate diagnosis) for ATAAD patients.

**Methods:**

From November 2004 to June 2020, of more than 200 patients with ATAAD patients who underwent aortic surgery at our hospital, there were 30 patients without pre-operative shock and malperfusion who sought for medical attention with symptoms for several days or delayed aortic surgery several days later despite ATAAD was diagnosed. Of the 30 patients (median age 60.9, range 33.4~82.5 years) in the study group, there were 18 patients undergoing surgery when they arrived at our hospital (delayed diagnosis by the patient) and 12 patients receiving surgery days later (delayed surgery despite immediate diagnosis). Patients with prompt surgery after symptom onset (control group) were matched from our database by propensity score matching. The surgical mortality rate and post-operative morbidities were compared between the study group and control group.

**Results:**

The in-hospital mortality was 3.3% for the study group and 6.7% for the control group (*p* = non-significant). The incidence of post-operative cerebral permanent neurological defect was 0% for the study group and 13.3% for the control group (*p* = 0.112). There were three patients receiving aortic re-intervention or re-do aortic surgery during follow-up for the study group and two patients for the control group.

**Conclusion:**

Prompt surgery for ATAAD is usually a good choice if everything is well prepared. Besides, urgent but non-prompt aortic surgery could also provide acceptable surgical results for ATAAD patients without pre-operative shock and malperfusion who did not seek medical attention or who could not make their minds to undergo surgery immediately after symptom onset. Hospitalization with intensive care is very important for pre-operative preparation and monitoring for the patients who decline prompt aortic surgery.

## Introduction

Acute type A aortic dissection (ATAAD) requires urgent surgical treatment (1, 2). If left untreated, it was claimed that the mortality rate could be 1–2% per hour during the first 24–48 h, and it could reach 75% at 2 weeks and 91% at 1 year (3). Therefore, aortic surgery is usually planned as soon as possible after ATAAD is diagnosed. However, urgent surgery is not equal to providing surgical treatment on an as-soon-as-possible basis. Moreover, despite improvement of surgical outcomes for ATAAD in the current era, surgical mortality rates for ATAAD remain high, with rates of 10–19% reported in the literatures (4–6). Therefore, the strategy of performing aortic surgery as soon as possible for all patients with ATAAD should be re-evaluated to determine whether modifications that could result in better clinical outcomes are possible. The majority of patients with ATAAD treated at our hospital underwent emergent aortic surgery. However, some ATAAD patients do not seek medical attention until several days after the onset of symptoms (delayed diagnosis caused by the patient), and some patients delay aortic surgery for several days despite ATAAD being diagnosed on the same day as symptom onset (delayed surgery despite immediate diagnosis). This study investigated the outcomes of ATAAD after non-prompt aortic surgery (delayed diagnosis caused by patient or delayed surgery despite immediate diagnosis).

## Patients and methods

**Figure 1:**
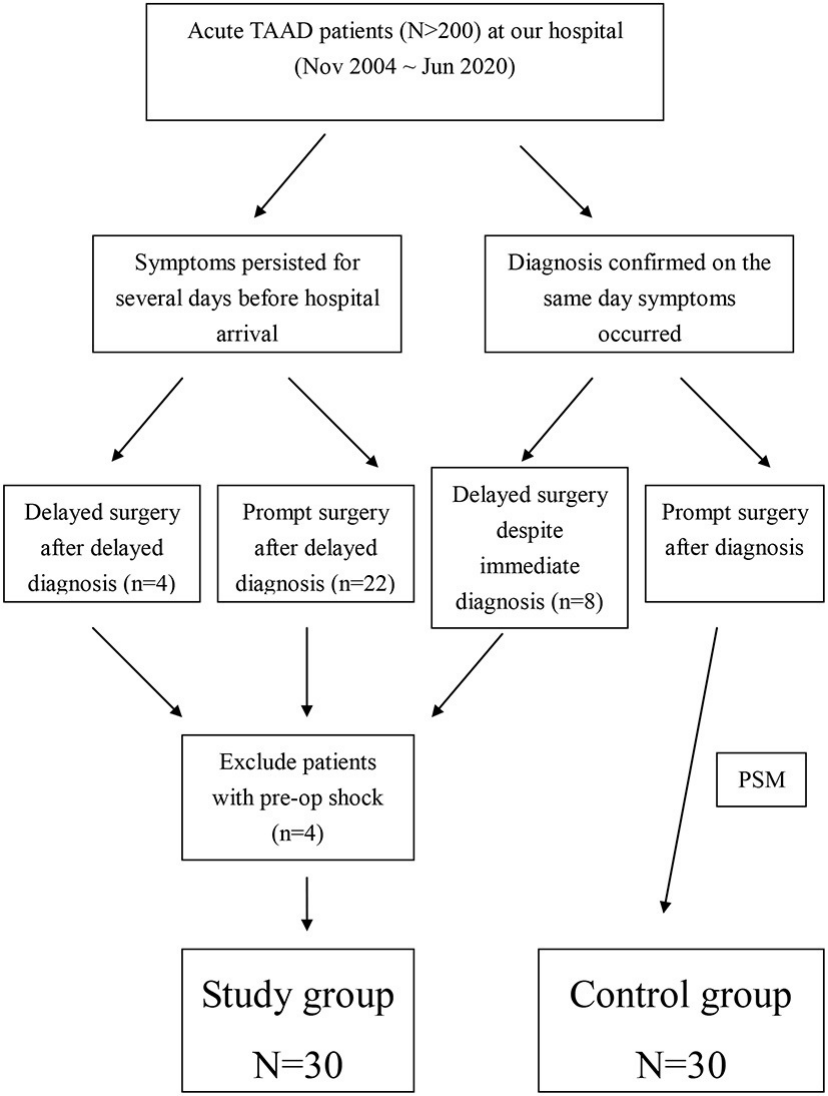
Grouping of the study group and the control group. PSM, propensity score matching.

From November 2004 to June 2020, more than 200 consecutive patients with ATAAD were treated surgically at our hospital. Among all ATAAD patients who underwent aortic surgery, 34 sought medical attention several days (median, 5 days; range, 3–7 days) after the onset of symptoms (chest pain, back pain, or shortness of breath) or delayed aortic surgery for several days (median, 3 days; range, 2–7 days) after the diagnosis of ATAAD even though it was diagnosed on the same day as the onset of symptoms (Figure 1). All patients were diagnosed using computed tomography (CT). Patients with preoperative shock (*n* = 4) were excluded from this study because it has a strong impact on the decision-making of the patients and their family. For all ATAAD patients who presented to our emergency department, intensive care unit (ICU) admission was required to monitor patients who were reluctant to undergo surgery immediately despite the recommendation of surgical treatment by the surgeons and patients who did not undergo immediate surgery as judged by the surgeons. For the patients (*n* = 30) who did not receive aortic surgery promptly after symptom onset, all got symptoms relieved and vital signs were stabilized after management in the emergency department. During hospitalization in the ICU, some patients experienced clinical progression. At that time, they changed their minds and decided to undergo aortic surgery. Aortic rupture did not occur pre-operatively because these patients were intensively monitored in the ICU. Of the 30 patients (mean age, 58.94 ± 12.81 years; median age, 60.9 years; range, 33.4–82.5 years) without preoperative shock and malperfusion, 26 (86.7%) had typical ATAAD; however, 4 (13.3%) patients were diagnosed with type A aortic intramural hematoma (IMH). Six patients (20%) were older than 70 years.

Because of the lack of randomization, and to reduce any selection bias, propensity score matching (PSM) was performed to match the study group patients with the control group patients from our database (Figure 1). The included patients did not experience preoperative shock and were treated at our hospital during the study period (November 2004 to June 2020). The parameters used for PSM included age, sex, hypertension, diabetes mellitus, hyperlipidemia, history of cerebrovascular accident (CVA), chronic renal insufficiency (CRI), chronic obstructive pulmonary disease (COPD), history of coronary artery disease (CAD), pre-operative shock, pre-operative acute myocardial infarction (AMI), and pre-operative CVA. Patients in the study group and control group were matched 1:1 using nearest neighbor PSM without replacement. The matched demographic data and preoperative conditions (*n* = 30 in both groups) are listed in Table 1.

**Table 1:** Characteristics of all patients with type A aortic dissection: study group (non-prompt surgery after symptom onset; control group: prompt surgery after symptom onset).

**Characteristics**	**Study group (delayed op after s/s onset)**					**Control group (*n* = 30)**	**PSCG**	**Standardized difference**	***P*-value#**
	**PSDD (*n* = 18)**	**DSID + DSDD (*n* = 12)**	*P*-value*	**Total (*n* = 30)**	**PSSG**				
Age (year)	62.71 ± 11.74 (33.4–82.5)	53.29 ± 12.72 (34.9–72.6)	0.054	58.94 ± 12.81 (33.4~82.5)	0.214	60.95 ± 13.40 (26.7~78.6)	0.210	0.15	0.552
Age (year) > 70	5 (27.8%)	1 (8.3%)	0.358	6 (20.0%)	0.292	9 (30.0%)	0.260	0.21	0.552
Gender (male)	9 (33.3%)	9 (75.0%)	0.260	18 (60.0%)	0.205	22 (73.3%)	0.200	0.23	0.412
Hypertension	15 (83.3%)	12 (100%)	0.255	27 (90.0%)	0.215	24 (80.0%)	0.205	−0.34	0.472
Diabetes mellitus	4 (22.2%)	2 (16.7%)	1.000	6 (20.0%)	0.355	3 (10.0%)	0.341	−0.29	0.472
Hyperlipidemia	6 (33.3%)	0 (0%)	0.057	6 (20.0%)	0.268	4 (13.3%)	0.277	−0.19	0.731
CVA history	2 (11.1%)	0 (0%)	0.503	2 (6.7%)	0.032	4 (13.3%)	0.096	0.21	0.671
CRI	1 (5.6%)	1 (8.3%)	1.000	2 (6.7%)	0.273	2 (6.7%)	0.273	−0.01	1.000
COPD	1 (5.6%)	1 (8.3%)	1.000	2 (6.7%)	0.525	2 (6.7%)	0.525	−0.01	1.000
CAD history	5 (27.8%)	1 (8.3%)	0.358	6 (20.0%)	0.428	4 (13.3%)	0.373	−0.19	0.731
Pre-op AMI	0 (0%)	0 (0%)	1.000	0 (0%)	–	0 (0%)	–	–	1.000
Pre-op CVA	1 (5.6%)	2 (16.7%)	0.548	3 (10.0%)	0.411	2 (6.7%)	0.266	−0.13	1.000

Values are presented as mean ± standard deviation (range) or n (%). s/s, symptoms and signs; CVA, cerebrovascular accident; CRI, chronic renal insufficiency; COPD, chronic obstructive pulmonary disease; CAD, coronary artery disease; AMI, acute myocardial infarction; CPR, cardiopulmonary resuscitation, *comparison between the 2 subgroups in the study group; #comparison between the study group and the control group; PSDD, prompt surgery after delayed diagnosis; DSID, delayed surgery despite immediate diagnosis; DSDD, delayed surgery after delayed diagnosis; PSSG, propensity score for study group; PSCG, propensity score for control group.

In the study group (*n* = 30), 18 patients delayed the ATAAD diagnosis (median, 5 days; range, 3–7 days) but promptly underwent aortic surgery, four patients delayed the ATAAD diagnosis (median, 5 days; range, 3–7 days) and delayed aortic surgery (median, 2 days; range 2–7 days), and eight patients were diagnosed with ATAAD on the same day as symptom onset but delayed aortic surgery for several days (median, 3 days; range 2–7 days). Patients in the control group (prompt surgery after symptom onset) matched using PSM were immediately diagnosed with ATAAD within hours after symptoms occurred and underwent surgery promptly after diagnosis. In addition to the comparison of the surgical outcomes between the study group and control group, the intra-group comparison of the surgical outcomes was also performed for the study group according to different surgical timing. The results of the intra-group and inter-group comparisons were shown in Tables 1–3.

**Table 2:** Intra-operative data.

**Characteristics**	**Study group**				**Control group (*n* = 30)**	**Standardized difference**	***P*-value#**
	**PSDD (*n* = 18)**	**DSID + DSDD (*n* = 12)**	*P*-value*	**Total (*n* = 30)**			
**Aortic lesion**							
Dissection	18 (100%)	8 (66.7%)	<0.001	26 (86.7%)	29 (96.7%)	0.37	0.353
IMH	0 (0%)	4 (33.3%)		4 (13.3%)	1 (3.3%)		
**CPB data**							
CPB time (min)	244 ± 49 (168~382)	253 ± 38 (207~313)	0.381	248 ± 45 (168~382)	201 ± 62 (117~420)	−0.85	0.002
AXC time (min)	157 ± 45 (86~292)	141 ± 29 (104~201)	0.301	149 ± 37 (86~292)	120 ± 41 (58–197)	−0.75	0.006
CA time (min)	63 ± 45 (56~179)	54 ± 17 (50~81)	0.500	59 ± 37 (50~179)	44 ± 16 (40~73)	−0.54	0.040
**Intra-operative cerebral protection**							
ACP	2 (11.1%)	2 (16.7%)	1.000	4 (13.3%)	3 (10.0%)	−0.11	1.000
RCP	16 (89.9%)	10 (83.3%)		26 (86.7%)	27 (90.0%)		
**Main procedures for aortic lesions**							
AAoG ± AVPR	12 (66.7%)	8 (66.7%)	1.000	20 (66.7%)	26 (86.7%)	0.48	0.125
HAR ± AVPR	5 (27.8%)	3 (25.0%)	1.000	8 (26.7%)	2 (6.7%)	−0.55	0.080
TAR ± AVPR ± FET	1 (5.6%)	0 (0%)	1.000	1 (3.3%)	1 (3.3%)	−0.51	1.000
Bentall	0 (0%)	1 (8.3%)	0.400	1 (3.3%)	1 (3.3%)	−0.51	1.000

CPB, cardiopulmonary bypass; AXC, aortic cross clamping; CA, circulatory arrest; AAoG, grafting of ascending aorta; AVPR, aortic valve repair or replacement; HAR, hemi-arch replacement; TAR, total arch replacement; FET, frozen elephant trunk; *comparison between the 2 subgroups in the study group; #comparison between the study group and the control group; PSDD, prompt surgery after delayed diagnosis; DSID, delayed surgery despite immediate diagnosis; DSDD, delayed surgery after delayed diagnosis.

**Table 3:** Post-operative data.

**Characteristics**	**Study group**				**Control group (*n* = 30)**	**Standardized difference**	***P*-value#**
	**PSDD (*n* = 18)**	**DSID + DSDD (*n* = 12)**	*P*-value*	**Total (*n* = 30)**			
**Surgical survival**							
30-day mortality rate	1 (5.6%)	0 (0%)	1.000	1 (3.3%)	1 (3.3%)	0.14	1.000
In-hospital mortality rate	1 (5.6%)	0 (0%)	1.000	1 (3.3%)	2 (6.7%)	0.14	1.000
**Post-operative PND**							
Post-operative cerebral stroke	0 (0%)	0 (0%)	–	0 (0%)	4 (13.3%)	0.35	0.112
Post-operative paraplegia	1 (5.6%)	0 (0%)	1.000	1 (3.3%)	0 (0%)	−0.26	1.000
**Hospital stay**							
All patients	19 ± 6 (9–27)	33 ± 19 (16–85)	0.026	27 ± 16 (9–85)	23 ± 12 (6–64)	−0.31	0.255
Hospital survivors	33 ± 20 (16–85)	19 ± 6 (9–27)	0.021	27 ± 16 (9–85)	23 ± 12 (12–64)	−0.31	0.263
Hospital non-survivors	23	–	–	23	22 ± 23 (6–38)	–	–
**Long-term survival**							
Followed-up duration (year)	5.0 ± 4.5 (0.1–13.6)	2.9 ± 1.9 (0.6–6.3)	0.351	4.2 ± 3.8 (0.1–13.6)	7.5 ± 6.1 (0.1–19.3)	0.66	0.013
5-year survival	80.8%	100%	0.166	87.5%	93.6%	–	0.221
10-year-survival	70.7%	–		77.8%	85.8%	–	

PND, permanent neurological deficits; *comparison between the 2 subgroups in the study group; #comparison between the study group and the control group; PSDD, prompt surgery after delayed diagnosis; DSID, delayed surgery despite immediate diagnosis; DSDD, delayed surgery after delayed diagnosis.

### Operative procedures

The chest was entered by median sternotomy and the pericardium was opened after the cardiopulmonary bypass (CPB) circuit was prepared. One femoral artery, right axillary artery, or ascending aorta was explored for arterial cannulation, and the superior vena cava and inferior vena cava or one femoral vein were used for venous cannulation for the CPB under general endotracheal anesthesia. For the patients with direct aortic cannulation (7), one Fr23 straight arterial cannula (Maquet, Germany) was inserted using Seldinger's technique. Epi-aortic sonography was performed to evaluate the puncture site, and trans-esophageal echocardiography was performed to confirm the proper position of the arterial cannula. Purse-string sutures might be necessary to secure the arterial cannula inserted in the ascending aorta. After cannulation was completed, CPB and systemic cooling were started. For all the patients in the study group and control group, antegrade or retrograde cerebral perfusion was performed as an adjunct for cerebral protection during circulatory arrest according to the surgeon's preference. For retrograde cerebral perfusion (RCP), systemic perfusion was stopped when the core body temperature reached 18°C and retrograde cerebral perfusion (10–12 mL/kg) was administered through the internal jugular vein; however, for antegrade cerebral perfusion (ACP), blood flow (10 mL/kg) to the brain was infused through the right axillary artery when the core body temperature was 25°C. Ice was packed around the head of all patients during the entire circulatory arrest period. Cerebral oximetry with near-infrared spectroscopy was used intraoperatively to estimate the oxygen saturation within the tissues of the frontal lobe of the brain. Plegisol (Pfizer, USA) or HTK (histidine-tryptophan-ketoglutarate) solution (Custodiol, Germany) was used as the cardioplegic solution directly infused in the ostium of the left coronary artery and right coronary artery after aortotomy. Surgical procedures included ascending aortic grafting (AAoG), AAoG with aortic valvuloplasty (AVP), AAoG with aortic valve replacement (AVR), AAoG with AVP and coronary arterial bypass grafting (CABG), AAoG with AVR and CABG, AAoG with mitral valve replacement (MVR) and CABG, Bentall procedure, hemi-arch replacement (HAR), HAR with AVP, total arch replacement (TAR), and TAR with frozen elephant trunk (FET). The procedure was chosen according to the judgment of the surgeons. HAR was usually chosen for the intima tear site when it was near or in the proximal aortic arch. TAR was usually performed for the intima tear site when it was in the aortic arch. Bentall procedure was used for Marfan syndrome or annuloaortic ectasia. For other cases, ascending aortic grafting was performed. The ascending aorta or both the ascending aorta and aortic arch were resected. Then, open anastomosis was performed for aortic grafting using the sandwich technique, whereby both ends of the aorta were reinforced with Teflon felt strips. Tissue glue was used for hemostasis of the anastomosis sites between the aorta and vascular graft. Intra-operative data are summarized in Table 2.

Regarding aortic regurgitation, it was not always necessary to ensure whether aortic regurgitation existed pre-operatively. If detachment of the commissures of aortic valve was observed intra-operatively, then re-suspension of the aortic valve was usually performed with aortic grafting. However, aortic valve replacement was another option.

### Statistical analysis

Continuous variables are presented as the mean ± standard deviation (SD) or median and range. Categorical variables are expressed as numbers and percentages. PSM was performed to match the study group with the control group, and the standardized difference was calculated. Continuous variables of the study group and control group were compared using the Student *t*-test, and the Mann-Whitney *U* test was used to compare continuous variables of the two subgroups in the study group. Categorical variables were compared using the chi-square test or Fisher's exact test. The Kaplan-Meier method with log-rank testing was performed to compare the long-term survival of the study group and control group. *P* < 0.05 was considered statistically significant. Statistical analyses were performed using STATA statistical software (STATA Corp, College Station, TX, USA).

## Results

There were no significant differences in the baseline characteristics of the study group and control group because PSM was applied. In the study group, there was a trend of older patients and more patients with hyperlipidemia who were willing to promptly undergo surgery.

Of the patients in the study group (*n* = 30), 20 patients underwent grafting of the ascending aorta (six AVP, four AVR, two CABG, one TVP concomitantly), eight underwent HAR (three AVP concomitantly), one underwent TAR with FET (stent graft) and one underwent Bentall procedure. Of the patients in the control group (*n* = 30), 26 underwent grafting of the ascending aorta (13 AVP, one AVR, one MVR, one CABG concomitantly), two underwent HAR (one AVP concomitantly), one underwent TAR with FET (stent graft) and one underwent Bentall procedure.

Regarding the strategies of cerebral protection during aortic surgery, 26 patients underwent RCP and four patients underwent ACP in the study group; in the control group, 27 patients underwent RCP and three patients underwent ACP.

The mean CPB time was 248 ± 45 min (range 168–382 min) in the study group and 201 ± 62 min (range 117–420 min) in the control group. The aortic cross-clamping time was 149 ± 37 min (range 86–292 min) in the study group and 120 ± 41 min (range 58–197 min) in the control group. The circulatory arrest time was 59 ± 37 min (range 50–179 min) in the study group and 44 ± 16 min (range 40–73 min) in the control group. The mean CPB time, aortic cross-clamping time, and circulatory arrest time were shorter in the control group than in the study group because there were more patients with AAOG in the control group than in the study group.

The in-hospital mortality rates were 3.3% (1 of 30 patients) in the study group and 6.7% (2 of 30 patients) in the control group. There was no statistically significant difference. The cause of in-hospital mortality for one patient (age, 66.5 years) in the study group was multi-organ failure. The causes of death in the control group were cardiac failure for one patient, and central nervous system failure caused by right cerebral ischemic stroke for one patient.

Regarding the postoperative neurological deficits (PNDs), all patients in the study group regained consciousness after aortic surgery, but one patient (3.3%) in the study group experienced postoperative paraplegia that did not seem related to aortic surgery; the exact etiology of the spinal cord ischemia was unclear. Four patients (13.3%) in the control group experienced an ischemic cerebral stroke after aortic surgery.

Additionally, other major post-operative complications included debridement for sternal wound infection for one patient in the study group, tracheostomy for one patient in the study group, re-wiring of the sternum for one patient in the control group, and debridement for sternal wound infection for one patient in the control group. No patients required re-sternotomy for mediastinal bleeding after aortic surgery.

The mean hospital stay was 27 ± 16 days (range 9–85 min) for the study group; however, it was 23 ± 12 days (range 6–64 min) for the control group. There was no statistically significant difference in the hospital stay.

**Figure 2:**
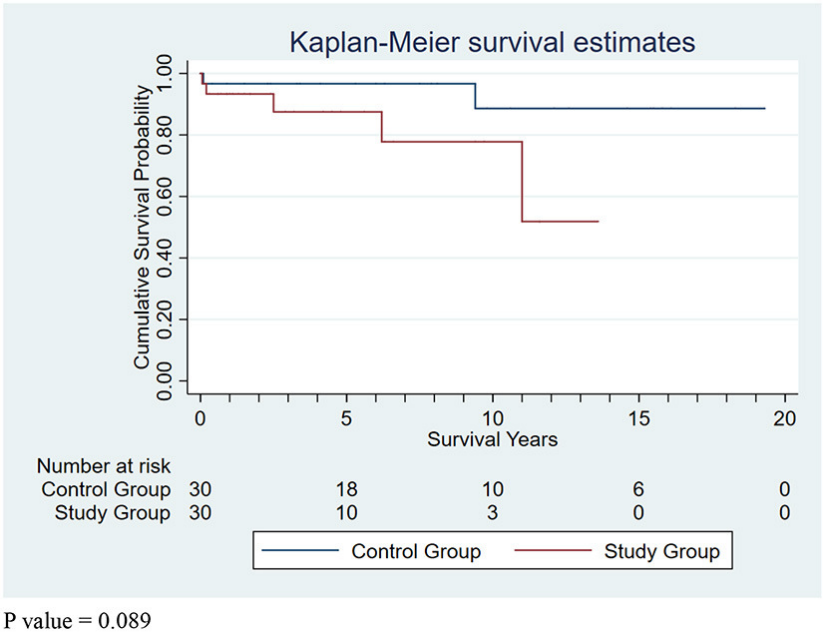
Kaplan-Meier survival curves for study group and control group. *P*-value = 0.089.

Regarding aortic re-intervention and re-do aortic surgery, the following procedures were performed: endovascular stent grafting for aortic arch aneurysm for one patient at 10.8 years later (died of central nervous system failure during admission for re-intervention) in the study group; endovascular stent grafting for contained rupture of the descending aorta for one patient at 5.1 years later in the study group; endovascular stent grafting for descending aortic aneurysm for one patient at 5.5 years later in the study group; open surgical grafting for descending aortic aneurysm for one patient at 11.3 years later in the control group; and total descending aortic grafting for descending aortic aneurysm for one patient with Marfan syndrome at 9.3 years later in the control group. Regarding the mortality for aortic re-intervention and re-do aortic surgery, only one patient who underwent endovascular stent grafting for aortic arch aneurysm died of central nervous system failure. Regarding long-term survival, the mean follow-up period was 4.2 ± 3.8 years (range 0.1–13.6 years), and the 5- and 10-year survival rate were 87.5 and 77.8% for the study group respectively. In the control group, the mean follow-up period was 7.5 ± 6.1 years (range 0.1–19.3 years), and the 5- and 10-year survival rate were 93.6 and 85.8%, respectively. The mean follow-up period was shorter in the study group than in the control group (*p* = 0.013); however, there was no statistically significant difference in long-term survival (Figure 2) between the study group and control group according to the log-rank test (*p* = 0.221). There was no statistically significant difference in the follow-up period and long-term survival of the two subgroups with non-prompt aortic surgery.

## Discussion

The surgical mortality rate for ATAAD is still relatively high (10–19%), although the surgical results are improving in the current era (4–6). Regarding the concerning correlation between clinical outcomes and the elapsed time since symptom onset for ATAAD patients, Booher et al. (8) used data (*n* = 1,160) from the Registry of Acute Aortic Dissection (IRAD) and found that the mortality rate was higher within the first 24 h after symptom onset. However, Inoue et al. (6) used data (*n* = 1,217) from the Japanese Registry of Aortic Dissection (JRAD) and found that the in-hospital mortality rate of patients transferred to JRAD hospitals within 150 min of symptom onset was statistically significantly higher than that of patients transferred after 150 min of symptom onset (16 vs. 8.8%; *p* < 0.01). Their findings suggested no consistent correlation between mortality and elapsed time after ATAAD symptom onset. However, their study (6) also showed that other factors, such as older age, preoperative shock, conscious disturbance, and cardiac arrest, had stronger impacts on in-hospital mortality. Moreover, Sabe et al. (9) proposed that the optimization of surgical outcomes requires thoughtful patient selection. Furthermore, the timing of surgery requires nuanced characterization of the severity and extent of dissection and potential reversibility of malperfusion syndrome (9). Therefore, the elapsed time from symptom onset to aortic surgery might be not an absolute independent factor associated with the survival of ATAAD patients, and other parameters might have a more important influence on the surgical mortality for ATAAD patients, such as frailty, preoperative shock, preoperative CPR, preoperative CVA, and chronic renal insufficiency (6, 9, 10). Therefore, although urgent aortic surgery for ATAAD patients is recommended by the current guidelines, the policy of performing aortic surgery as soon as possible seems sufficient for most, but not all, ATAAD patients. Deeb et al. (11) reported that the surgical mortality rate of patients who underwent delayed aortic replacement was statistically significantly better than that of ATAAD patients with malperfusion who underwent immediate aortic replacement (25 vs. 89%; *p* = 0.003). According to the JRAD (6), the in-hospital mortality rates were 16.6% for medically treated ATAAD patients (*n* = 301) and 10.8% for the surgical group (*n* = 916); furthermore, the 2-year survival rates of medically and surgically treated patients were not statistically significant (93 vs. 89%; *p* = 0.606) (6). This means that acceptable mid-term survival could be achieved for selected ATAAD patients who could not or did not undergo aortic surgery for various reasons, including advanced age, cardiac arrest necessitating resuscitation, shock with severe comorbidities, neurologic dysfunction, extremely stable condition, and thrombosed false lumen at the ascending aorta. However, it cannot be said that patients with the aforementioned parameters are not suitable for aortic surgery; rather, the importance of risk stratification and thoughtful communication with the patients to decide the best treatment strategy to achieve higher survival rates irrespective of prompt surgery, delayed surgery, or medical treatment must be emphasized (6, 12). On the other hand, Aoyama et al. (13) reported that the in-hospital mortality rate with medical treatment (31.6%) was almost twice that of patients who received surgical treatment (16.7%). Booher et al. (8) reported that the 14-day mortality rates were 18% for patients who received medical treatment and 8% for patients who received surgical treatment. Both studies revealed that although the short-term outcomes of surgical treatment were superior to those of medical treatment, the majority of patients could survive regardless of whether they received surgical or medical treatment. Emergency surgery was not an absolute guarantee of survival for all ATAAD patients, and medical treatment could stabilize the condition of most patients during the short term. Therefore, urgent, but not prompt, aortic surgery could be a possible option for selected ATAAD patients who could have more chance to survive the surgery performed by more experienced surgical teams and well-prepared facilities. Surgeons must pursue better survival for every patient; they should not base their decisions on statistical data alone.

Therefore, the policy of performing aortic surgery as soon as possible should be re-evaluated. It would be better to decide whether, where, and when to perform aortic surgery based on the condition of ATAAD patients instead of the anatomical classification of aortic dissection alone. For example, for the ATAAD patients with an extremely stable condition, immediate aortic surgery does not seem mandatory if the surgeon is inexperienced or if the facility is not prepared. It could be reasonable and practicable to transfer ATAAD patients with an extremely stable condition to an experienced center or wait for the support of experienced surgeons.

Regarding the timing of aortic surgery for ATAAD patients, Sabe et al. (9) proposed an algorithm to treat ATAAD patients; they reported that not all ATAAD patients should be treated surgically promptly after the diagnosis of ATAAD (9), and that there are some parameters that should be considered, such as hemodynamic instability, malperfusion syndrome of visceral organs or extremities, arterial obstruction necessitating endovascular treatment, aortic rupture, cardiac tamponade, and resolution of organ failure. This approach aims to ensure better survival for all ATAAD patients. Not all ATAAD patients achieve survival benefits with immediate aortic surgery. Therefore, risk stratification should be performed (14, 15), and the importance of patient stratification should be emphasized.

In our series, the patient (age, 66 years) who did not survive aortic surgery in the study group sought medical attention 2 days after symptom onset. Although aortic surgery was performed smoothly after the diagnosis of ATAAD, she died of multi-organ failure 23 days after surgery. All patients who delayed aortic surgery for several days because of various reasons despite ATAAD being diagnosed on the same days as symptom onset in the study group survived aortic surgery. This means that better surgical outcomes could be anticipated for ATAAD patients with an extremely stable condition even if the aortic surgery is not performed immediately. Regarding the influence on delayed surgery for aortic dissection progression in the aortic roots, such as aortic valve insufficiency and coronary compromise, we did not routinely perform echocardiography to evaluate the preoperative condition of the aortic valve in the emergency department because transesophageal echocardiography was usually performed to evaluate the aortic valve condition after anesthesia was administered for aortic surgery. We do not know if progression of aortic insufficiency or aortic dissection occurred in the study group patients awaiting aortic surgery. In our series, 12 patients underwent AVP or AVR and two patients underwent CABG in the study group; however, 15 patients underwent AVP or AVR and one patient underwent CABG in the control group. There was no significant difference between groups in terms of the surgical procedures for the aortic roots. Intensive monitoring should be performed for all ATAAD patients admitted to the hospital. Furthermore, surgeons should determine the optimal surgical timing (immediate or urgent but not prompt) based on the clinical condition of the ATAAD patients instead of performing immediate surgery routinely.

Another important issue for ATAAD patients who undergo aortic surgery is postoperative PND, which adversely affects the quality of life of survivors. In our series, no patients had postoperative cerebral PND and only one patient (2.9%) had postoperative spinal PND in the study group, even though the circulatory arrest time of the study group was longer than that of the control group (PND: 4 of 30; 13.3%). The cardiac surgical team is usually well-prepared for scheduled surgeries, and good surgical results can be anticipated. However, emergent scenarios may be different; for example, the members of the surgical team might be different, and other staff members supporting the emergent aortic surgery might not be very familiar with all the procedures. At our hospital, experienced surgeons perform aortic surgery. However, the residents assisting aortic surgeries during the day are more familiar with all the procedures than those who assist during the night. Surgical procedures performed at night might not be as smooth as those performed during the day, resulting in adverse outcomes. The performance of the assistants might adversely affect the surgical results despite the experience of the surgeons. Therefore, this is one of the reasons why to investigate the feasibility of non-prompt aortic surgery for ATAAD patients without preoperative shock who could achieve better surgical outcome. The strategy of urgent, but not prompt, aortic surgery for selected ATAAD patients without preoperative shock and malperfusion could have a chance to lower surgical mortality and postoperative PND.

## Study limitations

This was a retrospective study with a limited number of patients in the study group. A PSM analysis was performed to alleviate the selection bias and compare the clinical outcomes of the study group and control group. Large-scale studies should be performed to obtain further information and reach a final conclusion.

## Conclusion

It is important to carefully and thoroughly evaluate the clinical conditions of ATAAD patients and the hospital facilities before determining the treatment option and surgical timing. Prompt surgery for ATAAD is usually a good choice if the operators and the facility are well-prepared. Urgent, but not prompt, aortic surgery could provide acceptable surgical results for ATAAD patients without preoperative shock and malperfusion who did not seek medical attention in a timely manner or who did not agree to undergo surgery immediately after the onset of symptoms. For patients who decline prompt aortic surgery, hospitalization with intensive care is important for preoperative preparation and monitoring because these patients usually agree to undergo surgery when their symptoms become aggravated or their general condition deteriorates.

## Data availability statement

The raw data supporting the conclusions of this article will be made available by the authors, without undue reservation.

## Ethics statement

The studies involving human participants were reviewed and approved by MacKay Memorial Hospital Institutional Review Board, MacKay Memorial Hospital. Written informed consent for participation was not required for this study in accordance with the national legislation and the institutional requirements.

## Author contributions

S-JW: conception and design of study, acquisition of data, data analysis, drafting of manuscript, and approval of manuscript. Y-FF: acquisition of data, drafting of manuscript, and approval of manuscript. Y-CT: acquisition of data, data analysis, drafting of manuscript, and approval of manuscript. SS, C-YC, and J-YL: conception and design of study, drafting of manuscript, and approval of manuscript. All authors contributed to the article and approved the submitted version.

## Conflict of interest

The authors declare that the research was conducted in the absence of any commercial or financial relationships that could be construed as a potential conflict of interest.

## Publisher's note

All claims expressed in this article are solely those of the authors and do not necessarily represent those of their affiliated organizations, or those of the publisher, the editors and the reviewers. Any product that may be evaluated in this article, or claim that may be made by its manufacturer, is not guaranteed or endorsed by the publisher.
